# A space–time analysis of *Mycoplasma bovis*: bulk tank milk antibody screening results from all Danish dairy herds in 2013–2014

**DOI:** 10.1186/s13028-016-0198-3

**Published:** 2016-02-29

**Authors:** Margarida Arede, Per Kantsø Nielsen, Syed Sayeem Uddin Ahmed, Tariq Halasa, Liza Rosenbaum Nielsen, Nils Toft

**Affiliations:** 1 Section for Epidemiology, National Veterinary Institute, Technical University of Denmark, Bülowsvej 27, 1870 Frederiksberg C, Denmark; 2 Department of Large Animal Sciences, Faculty of Health and Medical Sciences, University of Copenhagen, Grønnegårdsvej 2, 1870 Frederiksberg C, Denmark

**Keywords:** Space–time analysis, *Mycoplasma bovis*, Mastitis, Dairy cattle, Denmark

## Abstract

**Background:**

*Mycoplasma bovis* is an important pathogen causing severe disease outbreaks in cattle farms. Since 2011, there has been an apparent increase in *M. bovis* outbreaks among Danish dairy cattle herds. The dairy cattle industry performed cross-sectional antibody screening for *M. bovis* on four occasions, using the indirect BIO K 302 *M. bovis* enzyme-linked immunosorbent assay (ELISA) (Bio-X, Belgium) in bulk tank milk from all dairy herds between June 2013 and July 2014. The objective of this study was to investigate the evolution of the spatial distribution of *M. bovis* in the Danish dairy herd population throughout the study period. Repeated bulk tank milk samples were used as a proxy for the herd-level diagnosis. Descriptive and spatial analyses were performed for the four screening rounds. Based on a previous diagnostic test evaluation study, the *M. bovis* status for each herd was determined as test-positive or test-negative using a cut-off of 50 optical density coefficient %. The spatial global clustering was evaluated through a modified K-function method, and local clusters were identified by scan statistics.

**Results:**

The results showed that *M. bovis* test-positive herds had a dynamic pattern in space. The global clustering analysis showed that *M. bovis* test-positive herds were spatially correlated in rounds one, three and four. These findings were supported to some extent by the local clustering analysis, which found significant high- and low-risk spatial clusters in rounds one and three in the north and south of the mainland.

**Conclusion:**

The clusters with a high risk of observing test-positive herds did not remain between sampling rounds, indicating that *M. bovis* did not tend to persist upon emergence in dairy herds. In contrast, the clusters with a low risk of observing test-positive herds persisted in the same area throughout the study period.

## Background

*Mycoplasma bovis* causes several production diseases in cattle, such as mastitis and arthritis [1]. Mastitis caused by *M. bovis* has been of increasing concern for farmers and veterinarians throughout the past decades, due to its negative impact on production and welfare. This pathogen is known to have an important economic impact due to the reduction in milk yield [2] and the increase in unplanned culling rates [2, 3]. Furthermore, the associated suffering and pain negatively affect animal welfare [4]. Its prevalence has been rising worldwide [5–7], but whether this is the result of a faster spread of the pathogen or a greater awareness of the pathogenic potential of this microorganism is unknown [3].

The primary route of *M. bovis* transmission is thought to be udder-to-udder in the milking parlour, though the spread of the bacteria to calves via the milk from infected cows, as well as direct contact between animals of all ages are also important transmission routes [1, 2]. The purchase of replacement heifers and cows (which are asymptomatic carriers of this agent) might account for the introduction of the disease and the origin of outbreaks [8]. Once the infection is established across different age groups in a herd, it can be difficult to eliminate [9]. Other factors counteracting the control and elimination of this disease from dairy herds [9] include: the lack of knowledge about *M. bovis* virulence factors and its mechanisms of pathogenesis [1, 4]; both natural and acquired resistance to most antibiotics in vivo [1, 10], and the absence of an effective vaccine.

The latest report on *M. bovis* herd-level prevalence in Danish dairy herds is out-dated [11]. Therefore, there is a current resurgence in research, due to reports of severe clinical outbreaks associated with this pathogen and the lack of current knowledge about the distribution of the infection in Danish cattle herds. Knowledge of possible space–time patterns of the disease at herd-level would be advantageous in the planning of a potential surveillance programme for *M. bovis*. This type of assessment has the potential to promote the establishment of control and prevention strategies by generating hypotheses of disease causation [12] and enables the planning of test-strategies, including choice of methods and testing frequencies.

Veterinary spatial and temporal epidemiology emerged in the late 1990s, after becoming very popular in the field of human disease epidemiology. The advances made within this area have facilitated the identification and adjustment for confounding factors and the development of new hypotheses regarding disease transmission by researchers and health officials [13]. To optimize spatial analysis, data should be analyzed using more than one technique [12]. This can be seen in many studies of different infectious diseases all over the world, for instance acute respiratory disease in cattle in Norway [14], Highly Pathogenic Avian Influenza (HPAI) in Bangladesh [15] and *Salmonella* Dublin in Denmark [16].

The objective of this study was to investigate the spatio-temporal patterns of *M. bovis* based on four available bulk tank milk (BTM) antibody screenings from all dairy cattle herds in Denmark in 2013–2014.

## Methods

### Sample collection

The Danish dairy cattle industry performed four full dairy herd population cross-sectional screenings of antibodies directed against *M. bovis* in BTM between 01 June 2013 and 01 July 2014, in order to estimate the apparent prevalence of *M. bovis* infection. Milk truck drivers collected the samples through the Danish milk quality control scheme, using standardized procedures. The farmers were not notified when the sampling would be performed. All samples were tested using the indirect BIO K 302 *M. bovis* ELISA test-kit (BIO-X Diagnostics, Jemelle, Belgium). Diagnostics were performed at the Eurofins Steins A/S Laboratory, Holstebro, Denmark. Based on a previous test-evaluation study, an optical density coefficient (ODC) ≥50 % was used to define test results from each herd as test-positive [17]. At that cut-off, the BTM ELISA was estimated to have a sensitivity (Se) = 43.5 % (95 % CI: 21.1–92.5 %) and specificity (Sp) = 99.6 % (95 % CI: 98.8–100 %).

Some herds were tested more than once per round because they participated in parallel projects or requested their own samples. However, only the sample with the highest ELISA-value in each round was kept in the dataset, as this was thought to improve the Se of the analysis without excessively reducing the Sp. All herds located on the island of Bornholm were excluded from the dataset, since their limited number and remote geographical location could introduce bias to the analysis.

Cartesian coordinates (EUREF 89; UTM zone N32) for all dairy herds included in the current study were available for spatial analysis.

### Spatial analysis

The spatial analysis of *M. bovis* test-positive herds in Denmark was accomplished in two steps: 1) the global spatial clustering was evaluated with the Monte Carlo simulation of the K-function [18]; 2) the local clustering was assessed using purely spatial scan statistics [19].

#### Global clustering

The K-function is a widely used method for evaluating global spatial clusters. The complete spatial randomness (CSR) defined by the absence of clustering is tested using a homogenous Poisson process for the null-hypothesis K-function. This assesses the global clustering of test-positive herds relative to the test-negative herds throughout the study region. The function does not identify the location of the clusters, instead it provides a summary of the spatial dependence between test-positive herds as a function of distance [20].

In order to overcome the assumptions connected with this technique and to adapt it to the present data, a Monte Carlo simulation of the K-function was applied [18]. The difference between the empirical K-function and the estimated null-hypothesis version of the K-function (the D-function), with 95 % confidence interval, was plotted against the distance between farms. This was done for each of the four sampling rounds.

#### Local spatial clusters

Local clusters were estimated with scan statistics [21] for each sampling round. This technique is characterized by a circular window, which is moved in space for each possible geographic location and size [19].

A Bernoulli model was applied in SatScan™ (version 9.4.1, Martin Kulldorff and Information Management Services Inc.; http://www.satscan.org/), where test-positive and test-negative herds were defined as previously described. The most likely clusters for higher or lower risk were identified by likelihood ratio testing, and their significance estimated through a Monte Carlo simulation consisting of 999 random replications of the dataset. A significance level of 5 % was used.

## Results

### Summary statistics

Of the total number of participating herds (3700), the majority were tested in all four rounds.

The overall decrease in the number of herds tested in each round throughout time reflects the demographic changes in the dairy herd population (Table 1).

**Table 1:** Descriptive data and apparent prevalence for each sampling round

Sampling round	1	2	3	4
Duration^a^	01 June–31 july 2013	01 August–31 december 2013	27 January–18 march 2014	11 June–01 july 2014
No of test-positive^b^	186	55	107	55
No of herds sampled^c^	3578	3583	3446	3379
Apparent prevalence^d^ (%)	5.2	1.5	3.1	1.6
(CI 95 %)^e^	(4.5–5.9)	(1.1–1.9)	(2.5–3.7)	(1.2–2.1)

^a^Duration of the sample period

^b^Number of test-positive herds for *M. bovis* in each sampling round

^c^Total number of sampled herds for *M. bovis* in each sampling round

^d^Apparent prevalence of *M. bovis* in each sampling round

^e^Confidence interval for the apparent prevalence of *M. bovis* in each sampling round

The prevalence of test-positive herds ranged from 1.6 to 5.2 % during the study period (Table 1).

During the first screening round, 186 herds were *M. bovis* test-positive. Of these, 179, 170 and 165 herds were also tested in rounds two, three and four, respectively, and only 18 (10 %), 28 (16 %) and 12 (7 %) retested positive for *M. bovis*.

The majority of the 55 test-positive herds (60 %) in the second round either tested negative or were not tested on the previous round. The third and fourth screening rounds showed a similar pattern, with 70 and 60 % of the test-positive herds for each round testing positive for the first time.

### Global clustering

**Fig. 1:**
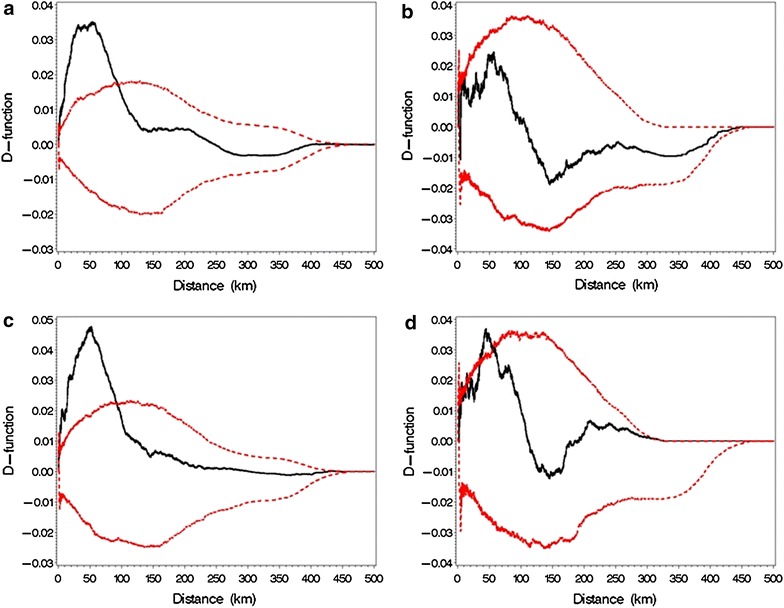
Global clustering per sampling round. Legend: estimated D-function (*black line*) with 95 % simulation envelope (*red line*) of *Mycoplasma bovis* test-positive herds for round 1 (**a**); round 2 (**b**); round 3 (**c**); and round 4 (**d**), in Denmark

The results of the D-function analyses for each round of sampling are illustrated in Fig. 1. The results indicate significant global clustering in the first and third sampling round, with the D-function rising above the 95 % simulation envelope in the first round at approximately 1–90 and 1–100 km in the third round. The analysis for the fourth round showed a decreased, yet significant global clustering with a modest rise in the D-function above the 95 % simulation envelope at a distance of approximately 40–60 km.

### Local clustering

**Fig. 2:**
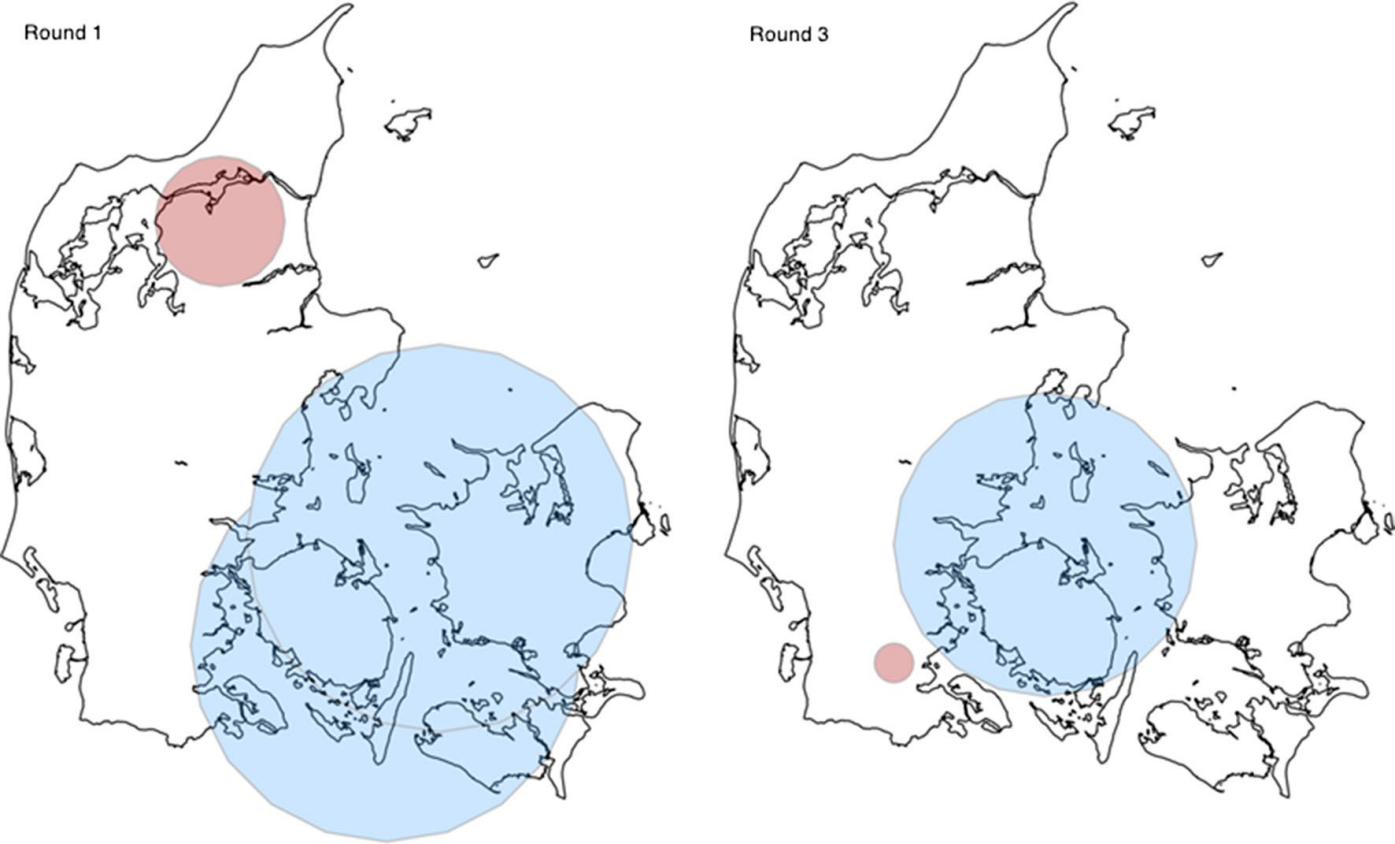
Map of significant local high-risk and low-risk clusters of *Mycoplasma bovis* infection in Denmark. Legend: location of the significant clusters with a high risk (*red closed circle*) and low risk (*blue closed circle*) of *M. bovis* test-positive herds in Denmark, by sampling round

The purely spatial analysis performed with different spatial windows of 50, 25 and 15 % of the population at risk showed consistency in size and location of the clusters. Results relating to the space scan-statistics for 15 % of the population at risk in each round are presented in Table 2. No significant low-risk or high-risk clusters were identified in rounds two or four. Figure 2 shows the location and size of the significant clusters in rounds one and three.

**Table 2:** Statistically significant spatial clusters of high- and low-risk *Mycoplasma bovis* test-positive herds in Denmark, by sampling round

Sampling round	Population^a^	Radius (km)	O^b^ (E^c^)	RR^d^	LR^e^	*P* ^f^
1	334	28.89	41 (17)	2.75	14.33	<0.010
	400	87.05	4 (20)	0.17	11.44	0.035
	356	85.93	3 (18)	0.15	11.13	0.047
3	43	8.73	10 (1)	8.15	12.81	0.011
	380	67.36	0 (11)	0	12.72	0.011

Results relating to the space scan-statistics for 15 % of the population at risk in each round are presented

^a^Number of herds in each cluster

^b^Observed number of test-positive herds in each cluster

^c^Expected number of test-positive herds in each cluster

^d^Relative risk

^e^Likelihood ratio

^f^ *P* value for the likelihood ratio test

The significant clusters with a high risk of test-positive herds were located to the north of the mainland for round one and to the south of the mainland for round three. The low-risk analysis of *M. bovis* test-positive herds identified two clusters in round one and one cluster in round three, all located in the southeast of Jutland, Funen and Zealand.

A density map of the average herd size of the sampled dairy herds during the study period was obtained using Quantum GIS.

## Discussion

The objective of this study was to explore the spatial distribution of *M. bovis* antibody-positive dairy cattle herds in Denmark and to identify temporal patterns and/or spatial persistence of test-positivity between screening rounds. The global cluster analysis showed that *M. bovis* test-positive herds were spatially correlated in screening rounds one, three, and (to a certain extent) four. These findings were confirmed by the local clustering analysis for rounds one and three, which identified significant spatial clusters: some of which were spatial clusters of herds with a higher risk of being test-positive, whilst the remaining clusters were herds with a lower risk than the other herds included in the analysis. The results for the purely spatial analysis are reliable as they were shown to be consistent throughout the different spatial windows.

**Fig. 3:**
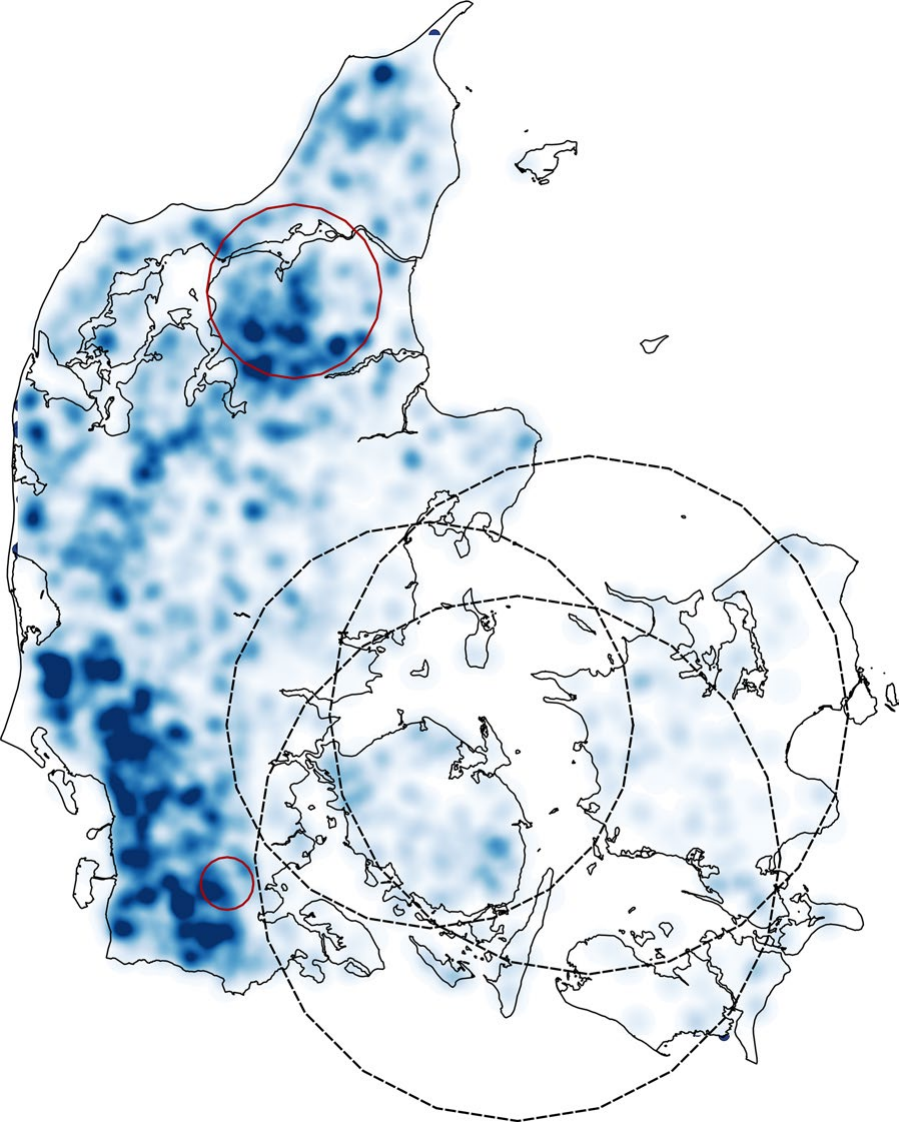
Density map of the average herd size of the dairy herds sampled during the study period. Legend: Location of the significant clusters with a high risk (*red solid line*) and low risk (*black dashed line*) of *Mycoplasma bovis* test-positive herds in Denmark

The local cluster analysis identified high-risk clusters of *M. bovis* test-positive herds to the north and south of the mainland, and low-risk clusters to the southeast of the mainland and islands, as well as central areas. Their locations might be partly explained by the different density of dairy cattle in these areas. The low-risk clusters were established in areas with a lower density of dairy cattle and the high-risk clusters were, to some extent, situated in areas where the density of dairy cattle was higher (Fig. 3). The higher animal turnover rate in larger herds (compared to smaller ones) is known to increase the risk of introducing an infected animal [6, 8]. This has been recognized both directly (by the positive association between presence of *Mycoplasma* sp. in BTM in larger herds [7, 22]) and indirectly (through the positive correlation between the weight of shipped milk per herd and the concentration of *M. bovis* in BTM [8]).

In rounds one and three, the low-risk clusters were located in approximately the same area, which implies the herds located in these regions had a lower risk of being infected by *M. bovis*. In contrast, the most significant clusters for the high-risk analysis differed markedly in location and size between screening rounds. The high-risk clusters were located to the north of the mainland in the first round and to the south of the mainland in the third round, with approximately 200 km between the two regions. This suggests that the occurrence of disease changed considerably in space throughout the study period (Fig. 2).

The cluster identified in the north in the first round was not present in the subsequent rounds. This implies a decrease in infected herds at this location in the months following the first round, assuming that a positive BTM ELISA test result indicates current or recent *M. bovis* infection. The cluster identified in round one contained 41 test-positive herds; of these, only eight herds (20 %) retested positive in the two following rounds, and only three herds (7 %) tested positive in the final round. With a similar pattern, the high-risk cluster identified in the third round had ten test-positive herds, of which only one herd (10 %) retested positive in the fourth round. In general, the test-positive herds in each round did not show a tendency to remain positive in subsequent rounds. In fact, in each round, at least 60 % of the test-positive herds were new. This suggests that the duration of infection in dairy herds is relatively short, an interpretation supported by Bray et al. [23], who found that *M. bovis* bacteria could not be detected in a herd 1 month after a positive diagnosis.

The growing awareness of *M. bovis* infection amongst farmers and local veterinarians might also explain the results indicating the short duration of herd infection [3, 7, 8]. According to these studies, measures such as culling infected cows with mastitis or reduced production, or isolating them on another farm with strict disease control procedures were very successful in limiting the spread of disease. Additionally, the self-limiting epidemiology of *M. bovis* could also be related to this cluster pattern. As Fox et al. [8] suggested, mastitis caused by *M. bovis* is associated with a brief period of high transmission that might be followed by a far lower transmission rate that is unable to maintain the presence of the pathogen in the mammary glands of a herd. It is possible that a small number of remaining infected animals excreting lower concentrations of bacteria (and hence producing lower levels of antibodies directed against *M. bovis*) might not be sufficient to elicit a response in the BTM ELISA above 50 ODC % (Petersen M, Krogh K, Nielsen LR; unpublished observations).

Taking into account what is known about *M. bovis* epidemiology, we can hypothesize how an animal may become infected with *M. bovis* in a herd turning test-positive between rounds. The infection could be introduced through a replacement heifer without quarantine or pre-movement testing, by exposure to other animals with pneumonia and arthritis caused by *M. bovis* [1, 2], or through auto-infection by a haematogenous route from other body sites to the mammary gland [6]. As stated previously, after an udder infection is established, the spread within the herd can occur quite rapidly. This is due to the large amount of bacteria shed in the milk before the onset of clinical mastitis infecting several other cows through udder-to-udder transmission at the milking parlour by the milking machines, teat cups or milkers’ hands [6].

Regarding the test outcome, false positive results are possible though unlikely, since the Sp of the test at the applied cut-off is close to 100 % [99.6 (95 % CI: 98.8–100)] [17]. False positive tests caused by carry-over between farms during sampling are thought to be negligible since the standardized sampling procedures should minimize this. The low Se of the ELISA test, 43.5 % (95 % CI: 21.1–92.5 %) at the applied cut-off, might have influenced the results and caused an underestimation of the infection. The analysis would probably have underestimated the size of the detected clusters and their significance, and/or have caused the analysis to miss smaller clusters. This could have been caused by dilution factors, intermittent shedding of *M. bovis* by chronically infected cows, or management practices such as withholding mastitic milk from the bulk tank [1, 4, 8, 22]. Furthermore, infection in young stock is not always detectable in the BTM (Petersen M, Krogh K, Nielsen LR; unpublished observations). It is, however, worth noting the large uncertainty of the Se estimate (95 % CI: 21.1–92.5 %), which is a consequence of the very low herd-level prevalence.

Although it was not possible to uncover a clear spatial and temporal pattern of the *M. bovis* infection in Denmark, we believe this is unlikely to be due to the data quality. The spatial distribution of the data is accurately represented since information about herd locations is available with no spatial aggregation. Some studies have data aggregated at polygon level and defined by administrative boundaries, which can lower the variance [16] and influence a false distribution pattern due to the selected boundaries [24]. Whilst in other studies, data are extracted from passive disease surveillance plans and only have information about cases, we had access to diagnostic information from a study population that contained the entire target population. However, the data presented drawbacks in sampling with an irregular timeframe and duration for each screening round.

Further investigation is required to study whether the *M. bovis* strains were the same between high–prevalence clusters in different rounds of sampling, as well as whether the pattern of animal movements between infected and uninfected herds or local short-distance spread (e.g., on pasture) can partially explain the existence of these clusters. It would also be of value to do the same analysis in the future, in order to assess whether the patterns throughout space and time would be similar to the ones reported by this study.

## Conclusions

There was no evidence for any *M. bovis* hotspots in Denmark, since the high-risk clusters of *M.bovis* test-positive herds appeared to have a short time span. However, it was verified that the low-risk clusters remained in the same location throughout time, indicating that herds in these geographical areas were at a lower risk of being test-positive for *M. bovis*. Nevertheless, further studies are needed to confirm this, and to elucidate the possible reasons as well as the implications for planning future control efforts.

## Abbreviations

BTM: bulk tank milk
CSR: complete spatial randomness
ELISA: enzyme-linked immunosorbent assay
*M. bovis*: *Mycoplasma bovis*
ODC: optical density coefficient
Se: sensitivity
Sp: specificity
UTM: universal transverse mercator coordinate system
